# Stability and Folding Behavior Analysis of Zinc-Finger Using Simple Models

**DOI:** 10.3390/ijms11104014

**Published:** 2010-10-19

**Authors:** Shan Chang, Xiong Jiao, Jian-Ping Hu, Yan Chen, Xu-Hong Tian

**Affiliations:** 1 College of Informatics, South China Agricultural University, Guangzhou 510642, China; E-Mail: ayan0426@scau.edu.cn; 2 Institute of Applied Mechanics and Biomedical Engineering, Taiyuan University of technology, Taiyuan 030024, China; E-Mail: jiaoxiong@emails.bjut.edu.cn; 3 College of Chemistry and Life Science, Leshan Teacher’s College, Leshan 614000, China; E-Mail: lion_hjp@yahoo.com.cn

**Keywords:** zinc finger, folding pathway, Gaussian network model, anisotropy elastic network model, Sp1f2, FSD-1

## Abstract

Zinc-fingers play crucial roles in regulating gene expression and mediating protein-protein interactions. In this article, two different proteins (Sp1f2 and FSD-1) are investigated using the Gaussian network model and anisotropy elastic network model. By using these simple coarse-grained methods, we analyze the structural stabilization and establish the unfolding pathway of the two different proteins, in good agreement with related experimental and molecular dynamics simulation data. From the analysis, it is also found that the folding process of the zinc-finger motif is predominated by several factors. Both the zinc ion and C-terminal loop affect the folding pathway of the zinc-finger motif. Knowledge about the stability and folding behavior of zinc-fingers may help in understanding the folding mechanisms of the zinc-finger motif and in designing new zinc-fingers. Meanwhile, these simple coarse-grained analyses can be used as a general and quick method for mechanistic studies of metalloproteins.

## 1. Introduction

How an amino acid sequence folds into a 3D model, that is the protein folding problem, is one of the fundamental and important questions in molecular biology. During the past few years, there were substantial improvements in the field of protein folding [1–7]. Also, some new and unclear problems arose, such as chaperonin-assisted and metal-coupled protein folding. Zinc-fingers are metalloproteins. Their folding needs the binding of zinc ion (Zn(II)), which is different from spontaneous protein folding. These proteins play crucial roles in regulating gene expression and mediating protein-protein interactions [8–11]. Understanding the folding and stability of zinc-fingers is fundamental for treating severe human diseases, for example cancer and neurological disorders. Meanwhile, knowledge of zinc-finger folding can provide insight into the general mechanisms of the metal-cofactor dependent protein folding [12–14] and also help developing better strategies for the metalloprotein design [15–17].

Some experimental and theoretical works have focused on identifying which factors affect the folding and stabilization of zinc-fingers and have revealed important features [2,9,18–20]. However, because of the limited temporal and spatial resolution in experiments, the detailed folding pathway data of the zinc-finger is still lacking. Recently, based on a quantum chemical method, Dudev and Lim predicted the binding order of the four conserved ligand-binding residues [21]. Wang *et al.* used the molecular dynamics (MD) method systematically to study the folding mechanisms of zinc-fingers [2,22,23]. They made the first simulation on the coupling between the metal binding and zinc-finger folding. In their simulations, both charge transfer and metal induced protonation/deprotonation effects have been studied. They found that zinc ion not only stabilizes the component secondary and tertiary structures, but also participates in the whole folding process of zinc-finger. Although some detailed molecular dynamics simulations have been performed for the zinc-fingers, the comparison studies of folding simulation are still a challenge for metalloprotein. Many contradictions still exist in previous studies of the zinc-fingers [22–26]. For example, the two different zinc-fingers, Sp1f2 and FSD-1, have similar structures, but their folding pathways produced by MD simulations are quite different [22,23]. Even though some simulations focused on the same proteins, they obtained different results by using different MD methods. By using an optimized atomistic model to the simulation of FSD-1, Kim *et al.* suggested that the folding is initiated by the collapse of the hydrophobic core and followed by the folding of the β-hairpin and the formation of the α-helix [24]. In comparison, Lei and Duan applied the all-atom MD model with AMBER force field to FSD-1 and they proposed that the folding of the individual secondary structures is prior to the formation of native hydrophobic core [25]. There were remarkable differences between the previous MD simulations. Therefore, it is necessary to analyze different zinc-fingers with the same simulation methods and some consistent mechanisms can be revealed through the comparisons. However, the all-atom MD simulation is time-consuming and computationally expensive, so comparison work is rarely reported. Meanwhile, the involvement of metal ions in the proteins makes the simulation much more complicated. Further, some special MD simulations [22,23,27] were applied to the zinc-finger, such as the replica exchange molecular dynamics (REMD) and molecular dynamics combined with quantum chemical calculations. These special MD methods are too professional to be applied to folding comparison of zinc-fingers by general researchers. Hence, developing a fast and simple simulation method becomes valuable for metalloprotein folding. In fact, the original zinc-finger has a different structure from the protein without zinc. Therefore, some simple model based on the structures [28–33] could provide useful information for zinc-finger folding.

In this work, we use the Gaussian network model (GNM) and anisotropy elastic network model (ANM) to analyze zinc-fingers. GNM is a schematic, coarse-grained model which is topology based and independent of sequence specificity [34–37]. GNM can provide information on conformational transition of proteins from the crystal structures and does not require the high computational cost of MD simulation. The ANM [38] is an extension of the GNM. Information about the directions of conformational transition can be obtained from analysis with ANM. Since the two classical elastic network models (ENMs) can provide the dynamic properties of proteins near an equilibrium state (usually native state), they are extensively applied to large systems that cannot be studied by all-atom MD, or applied to many proteins systematically for comparisons. However, the protein folding is usually far from the equilibrium state [3,39], so the general ENMs are not suitable for the protein folding study. Recently, some methods based on the iterative use of normal mode calculation were proposed to study protein folding/unfolding process [40,41]. These methods are the development of classical GNM, and the protein unfolding is considered to be approximated by a series of quasi-equilibrium processes corresponding to slowly increasing temperature. Su *et al*. [40] applied these methods to two classical small proteins, *i.e.*, CI2 and barnase. The iterative results obtained with the improved method were surprisingly in agreement with the MD simulation data. With a combination of classical and improved ENMs, our work may provide more comprehensive insight for the folding mechanisms of zinc-fingers.

In this article, the two different proteins, Sp1f2 [42] and FSD-1 [43], are analyzed by GNM and ANM. Our work shows how simple coarse-grained methods can investigate the mechanism of the zinc coupled folding of the zinc-finger motif. The coarse-grained methods only consider topological information and do not require the high computational cost of MD simulation. By using iterative GNM, we get the binding order of Zn(II) with the conserved ligand-binding residues and the unfolding pathway of Sp1f2 and FSD-1, which are all consistent with the experiment and MD simulation data. The previous MD simulations of the zinc-finger focused on the roles of zinc ion and the hydrophobic core, but the influence of C-terminal loop was rarely reported. In this work, we not only explain the function of zinc ion, but also discuss the effect of C-terminal loop. We find that both Zn(II) and the C-terminal loop affect the folding pathway of the different zinc-fingers. These are useful supplements for previous studies and can provide some insights into the design of zinc-fingers.

## 2. Systems and Methods

### 2.1. Protein Systems

**Figure 1:**
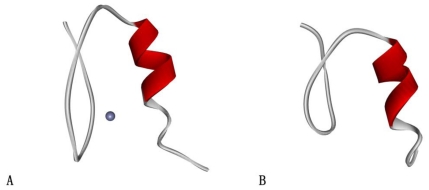
(**A**) Ribbon representation of the main-chain fold of Sp1f2 (PDB code: 1sp2); (**B**) Ribbon representation of the main-chain fold of FSD-1 (PDB code: 1fsd).

The two proteins were chosen for our analysis in this work are shown in Figure 1. One is the second finger of the human transcription factor Sp1 (Sp1f2), which is a typical Cys2His2 type zinc-finger (PDB code: 1sp2) [42] and has 31 residues (see Figure 1A). The other is a new protein FSD-1 (PDB code: 1fsd) [43], which contains 28 residues (see Figure 1B). FSD-1 is designed based on the classical zinc-finger and replaced the binding zinc with a larger hydrophobic core. This new protein is capable of folding to a similar structure as the original zinc-finger without the help of metal ions.

### 2.2. The Gaussian Network Model

In the Gaussian network model, a three-dimensional protein structure is described as an elastic network. In this work, both Cα atoms and Zn(II) are the vertices and they are connected by harmonic springs within a certain cutoff distance. The Zn(II) is encoded as 32 in the residue index of Sp1f2. The force constant is identical for all springs. Considering all contacting residues, the total energy of the network can be written as [35,44]

(1) $$V=\frac{1}{2}\gamma {\left\{\Delta R\right\}}^{T}\Gamma \{\Delta R\}$$

where *γ* is the harmonic force constant; {Δ*R*}represents the *N* dimensional vector whose elements are the fluctuation vectors Δ*R_i_* (*i* = 1,…,*N*) of the individual residues, where *N* is the number of residues. The superscript *T* denotes the transpose, and Γ is the *N* × *N* symmetric matrix. The elements in the symmetric matrix are written as

(2) $$\Gamma_{ij}=\{\begin{array}{llll} -1 & if & i\neq j\;and & R_{ij}\leq r_{c}\\ 0 & if & i\neq j\;and & R_{ij}>r_{c}\\ -\sum_{i,i\neq j}\Gamma_{ij} & if & i=j & \end{array}$$

where *R_ij_* is the separation between the *i*th and *j*th Cα atoms and *r_c_* is the cutoff distance (7.3 Å is adopted in this work).

The mean-square fluctuation of each atom and the cross-correlation fluctuations between different atoms are in proportion to the diagonal and off-diagonal elements of the pseudo-inverse of the Γmatrix. The inverse of the matrix can be decomposed as

(3) $$\Gamma^{-1}=U\Lambda^{-1}U^{T}$$

where *U* is an orthogonal matrix whose columns *u_k_* (1 ≤ *k* ≤ *N*) are the eigenvectors of Γ, and *Λ* is a diagonal matrix of eigenvalues *λ_i_* of Γ. The cross-correlation fluctuations between the *i*th and *j*th residues are given by

(4) $$<\Delta R_{i}\cdot \Delta R_{j}>\;=\frac{3k_{B}T}{\gamma }{\left[\Gamma^{-1}\right]}_{ij}$$

where *k_B_* is Boltzmann constant, *T* is absolute temperature. When *i* = *j*, the mean-square fluctuation of the *i*th residue can be obtained. The Debye-Waller or B-factor, which is related to the mean-square fluctuation, can be calculated with the expression

(5) $$B_{i}=8\pi^{2}\langle \Delta R_{i}\cdot \Delta R_{i}\rangle /3$$

In the GNM, the cross-correlation is normalized as

(6) $$C_{ij}=\frac{\langle \Delta R_{i}\cdot \Delta R_{j}\rangle }{{\left[\langle \Delta R_{i}^{2}\rangle \cdot \langle \Delta R_{j}^{2}\rangle \right]}^{1/2}}$$

### 2.3. The Iterative Unfolding Method

As the temperature of a protein is gradually increased, the native contacts between residues are expected to break in a fluctuation-dependent manner. The fluctuations in the distance between all residue pairs are calculated based on the GNM. The mean-square fluctuation in the distance vector *R_ij_* between the residues *i* and *j* can be written as [45]

(7) $$\begin{array}{l} \langle {\left(\Delta R_{ij}\right)}^{2}\rangle =\langle {\left(R_{ij}-R_{ij}^{0}\right)}^{2}\rangle =\langle {\left(\Delta R_{ij}-\Delta R_{j}\right)}^{2}\rangle \\ =\langle \Delta R_{i}\cdot \Delta R_{i}\rangle +\langle \Delta R_{j}\cdot \Delta R_{j}\rangle -2\langle \Delta R_{i}\cdot \Delta R_{j}\rangle \\ =\frac{3k_{B}T}{\gamma }({\left[\Gamma^{-1}\right]}_{ii}+{\left[\Gamma^{-1}\right]}_{jj}-2{\left[\Gamma^{-1}\right]}_{ij}) \end{array}$$

where *R_ij_* and *R_ij_* ^0^ are the instantaneous and equilibrium separation vectors between residues *i* and *j*.

The nonlinear elasticity during protein unfolding is considered through iterative normal mode calculations. As we know, the interaction between *i* and *i* ± 1 residues is the covalent bond, so only the noncovalent contacts between residue pairs can be broken in the iterative process. Then, the protein unfolding process is mimicked as the following scheme:

- The mean-square fluctuations of the distance in all noncovalent residue pairs are calculated based on the native structure topology with Equation 7.
- The noncovalent contact in the residue pair with the largest distance fluctuation is broken. Then, a new matrix Γ is obtained, which represents a new topology during protein unfolding.
- The mean-square fluctuations of the distance in all noncovalent residue pairs are recalculated based on the new matrix Γ using Equation 7.
- The above two steps are repeated until all the noncovalent contacts are broken.

Finally, all the topologies of different conformation during protein unfolding are obtained and the unfolding pathway can be derived from the data obtained above.

### 2.4. The Anisotropy Elastic Network Model

The GNM model can provide the amplitudes of residue fluctuations but no information about the directions of the fluctuations. Then, the ANM model [38] is introduced, by which information about the orientation of fluctuations is elicited. In ANM, the motion mode of a protein is determined by a Hessian matrix *H*.

(8) $$H=\left(\begin{array}{cccc} h_{11} & h_{12} & \cdots & h_{1N}\\ h_{21} & h_{22} & \cdots & h_{2N}\\ \vdots & \vdots & \vdots & \vdots \\ h_{N1} & h_{N2} & \cdots & h_{NN} \end{array}\right)$$

The elements of *H* are submatrix with size 3 × 3. The *ij*th submatrix *h_ij_* is

(9) $$h_{ij}=\left(\begin{array}{ccc} \frac{\partial^{2}V}{\partial x_{i}\partial x_{j}} & \frac{\partial^{2}V}{\partial x_{i}\partial y_{j}} & \frac{\partial^{2}V}{\partial x_{i}\partial z_{j}}\\ \frac{\partial^{2}V}{\partial y_{i}\partial x_{j}} & \frac{\partial^{2}V}{\partial y_{i}\partial y_{j}} & \frac{\partial^{2}V}{\partial y_{i}\partial z_{j}}\\ \frac{\partial^{2}V}{\partial z_{i}\partial x_{j}} & \frac{\partial^{2}V}{\partial z_{i}\partial y_{j}} & \frac{\partial^{2}V}{\partial z_{i}\partial z_{j}} \end{array}\right)$$

When *i ≠j*, the analytic expression for the elements of *h_ij_* is

(10) $$\frac{\partial^{2}V}{\partial x_{i}\partial y_{j}}={\frac{-\gamma (x_{j}-x_{i})(y_{j}-y_{i})}{R_{ij}^{2}}|}_{R_{ij}=R_{ij}^{0}}$$

When *i* = *j*, the analytic expression for the elements of *h_ij_* is

(11) $$\frac{\partial^{2}V}{\partial x_{i}\partial y_{j}}=\gamma {\sum_{j\neq i}\frac{\left(x_{j}-x_{i})(y_{j}-y_{i}\right)}{R_{ij}^{2}}|}_{R_{ij}=R_{ij}^{0}}$$

The meanings of *γ* and *R* are the same as that of Equation 1. *x*, *y*, and *z* represent the coordinates of atoms. In ANM, the cutoff distance *r_c_* is set as 13 Å.

## 3. Results and Discussion

### 3.1. Force Constants for Inter-Residue Interactions

**Figure 2:**
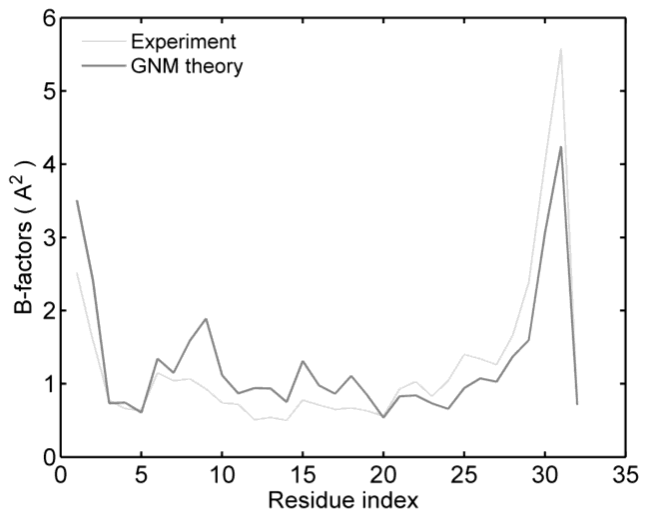
Experimental (light line) and calculated (dark line) B-factors of Sp1f2.

To evaluate the feasibility of the application of the GNM method in studying zinc-fingers, the B-factors are calculated and compared with the data from nuclear magnetic resonance (NMR). Setting *j* = *i* in Equation 4, we can obtain the mean-square fluctuations of residue *i*, <Δ*R_i_* Δ*R_i_*>. According to Equation 5, the B-factors of Sp1f2 are related to the mean-square fluctuation and calculated by using *B_i_* = 8*π*^2^<Δ*R_i_* Δ*R_i_*>/3. It can be seen, from the theory described above, that the only adjustable parameter in this work is *γ*. Then, *γ* can be determined by comparing the theoretically predicted mean-square fluctuations of Cα with those indicated by the experimental B-factors. The resulting *k_B_T*/*γ* value used for the zinc-fingers structure is 0.068 Ǻ^2^. Figure 2 shows the comparison between the experimental data of Sp1f2 (light line) and the calculated B-factor of Cα atoms (dark line), in which the correlation coefficient is 0.885 for Sp1f2. The results are similar to those of recent studies for other proteins [40,46].

### 3.2. The Fast Modes of the Motions

**Figure 3:**
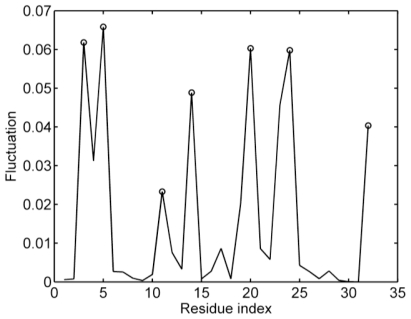
The fastest five mode shapes of the Sp1f2. There are several peaks marked in the curve that correspond to the kinetically key residues.

The fast modes of the motions correspond to the geometric irregularity in the local structure. Previous studies have found that high frequency fluctuating residues are thought to be kinetically key residues and critically important for the stability of the tertiary fold [47,48]. Figure 3 shows the fastest five modes of Sp1f2. In the structure of Sp1f2, the three residues (*i.e.*, Phe3, Phe14 and Leu20) are the conserved hydrophobic ones and the conserved ligand-binding residues are Cys5, Cys10, His23 and His27 [22]. As shown in Figure 3, Phe3, Cys5, Phe14, Leu20, Zn32 are the peaks in the curves. Meanwhile, the peaks Gly11 and Lys24 are close to the residues Cys10 and His23. Our results agree well with previous studies [22] and indicate that these residues play a key role in the stability of the protein. In the classical zinc-finger motif, the hydrophobic residue Phe3 is highly conserved. Wang *et al.* gave an explanation for the conservation [22]. They indicated that Phe3 is involved in the hydrophobic interactions with the nonpolar groups of the central loop region and stabilizing the N-terminal β-hairpin should be one of the possible reasons for nature to select this hydrophobic residue. Similarly, our results can also suggest that this hydrophobic residue is a key residue for the stability of Sp1f2, which may be another support for its conservation. Figure 3 also confirms that the Zn(II) is very important for the stability of the protein. However, the fluctuation of His27 is not high in the fast modes. His27 is in the long C-terminal loop of Sp1f2, which has large and global motions. Actually, in previous studies [21,22], it was also found that His27 is the last residue coordinating to the Zn(II) in the four conserved ligand-binding residues.

### 3.3. The Slow Modes of the Motions

**Figure 4:**
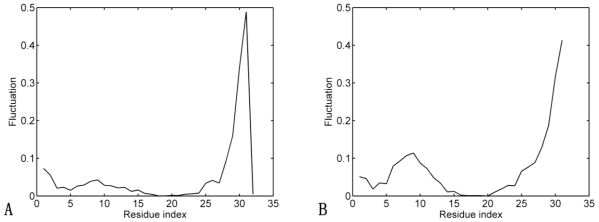
(**A**) The slowest mode shapes of Sp1f2 with zinc binding; (**B**) The slowest mode shapes of Sp1f2 without zinc binding.

The slow and long-wavelength collective modes usually represent functionally relevant motions of protein [48]. Figure 4 displays the slowest mode calculated by the GNM. From Figure 4, comparing the slowest modes of Sp1f2 with (holo-peptide) and without (apo-peptide) zinc binding, it can be seen that there are obvious differences between the two curves. As derived from Figure 4A, in the Sp1f2 with zinc binding, most of the residues are of low values of fluctuation. It means that these structures remain stable. However, in the apo-peptide structure (Figure 4B), the fluctuation values of residues Cys5 to Phe14 increase remarkably. The fluctuation increase of these residues implies that without zinc binding, β-hairpin becomes less stable in the apo-peptide structure than in the holo-peptide structure. It obviously confirms that Zn(II) is the key residue for the stability of β-hairpin. However, in the apo-peptide structure, the fluctuation values of residues Phe14 to His23 stay low. This can be explained by the packing of the hydrophobic Phe14, Leu20 and His23. These residues form the hydrophobic core, which is responsible for the formation and stabilization of Sp1f2 [49,50]. Meanwhile, there are some obvious increases for both structures at the end of the curve. It indicates that the C-terminal loop has global slow motions. This result can also confirm that His27 is not stable and the C-terminal loop may have an influence on the folding of Sp1f2.

### 3.4. Sequences of Unfolding Events of Sp1f2 and FSD-1 Revealed by Iterative Unfolding Method

Using the iterative unfolding method described above, the unfolding processes of Sp1f2 and FSD-1 are obtained. Based on this simple model, the results show that the two proteins have quite different folding pathways. Meanwhile, the order of the unfolding events is consistent with that of the thermal unfolding of proteins obtained with full atom MD simulation and experiments [22,23,51,52], which implies that the topology may play an important role in the two proteins folding process. This iterative unfolding method is coarse-grained and topology-based. Our results indicate that this simple model is an effectual method to explore the unfolding process of metalloprotein.

**Figure 5:**
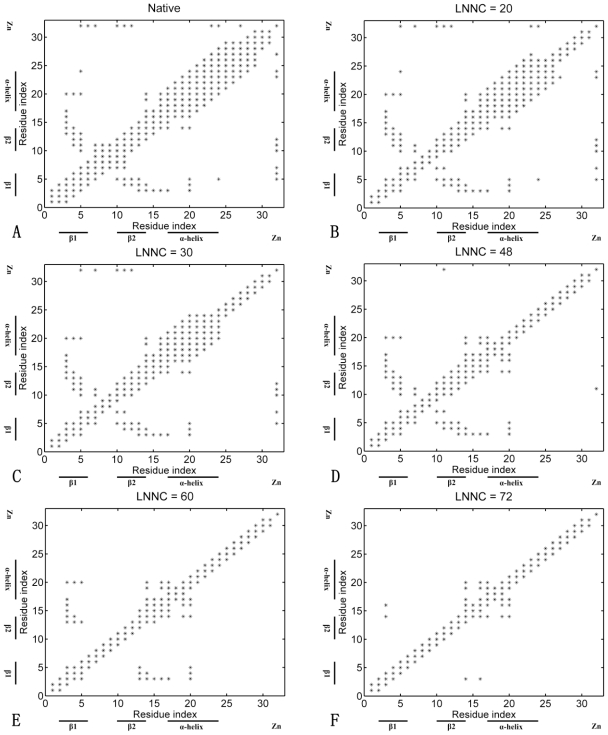
The contact maps of the native conformation (**A**), of the conformations with the LNNC to be 20 (**B**), 30 (**C**), 48 (**D**), 60 (**E**) and 72 (**F**) for Sp1f2, respectively. Each native contact is marked by the symbol * in the maps.

To elaborate the loss of the native contacts in the course of unfolding simulations, the contact maps of the conformation in different snapshots are constructed. Figure 5 presents the contact maps of the native structure (A), of the conformations with the loss-number-of- noncovalent-contact (LNNC) to be 20 (B), 30 (C), 48 (D), 60 (E) and 72 (F) for Sp1f2, respectively. The results reveal that there is a preferred process that shows a sequence of events for the unfolding of Sp1f2. The first unfolding event is the disappearance of the native contact in the C-terminal loop, as shown in Figure 5B. Then, the native contacts of the α-helix are partially lost, which implies that the unfolding of the C-terminal loop affect the stability of α-helix. During the partial unfolding of the α-helix, the bond between Zn(II) and His27 break first. Then, the loss of contacts between residues in β-hairpin is followed. Interestingly, as shown in Figure 5 (C, D), Zn(II) break the bond of Zn(II)-Cys5 before the bond of the Zn(II)-Cys10. This breaking order is in good agreement with the MD simulation [22] and experimental observation [51,52]. In addition, the folding cooperativity is considered as an important behavior of protein folding dynamics [53]. Previous studies [22] found that the zinc binding and the hydrophobic core formation are quite cooperative. In our model, the unfolding pathway is sequential and it is difficult to observe the cooperativity directly in the unfolding process. In fact, these highly cooperative behaviors take place in the near-neighbor steps for this iterative unfolding model. As shown in Figure 5B, when Cys5, Cys10 and His23 bind to Zn(II), the hydrophobic core is stable and integrated. After the breaking up of the Zn(II)-His23 bond, both of the hydrophobic core and the bonds between Zn(II) and two cysteines begin to unfold (see Figure 5C). When the bond of Zn(II)-Cys5 breaks, the native contacts of His23 are completely lost and the hydrophobic core is partly unfolding (see Figure 5D). These two behaviors happen in the near-neighbor phases and they may show cooperation to some extent. At the end of unfolding (see Figure 5 (E, F)), the native contacts of β-hairpin disappear and only α-helix is partially retained. This unfolding pathway is excitingly consistent with the pathway of MD simulation [22]. The results indicate that the unfolding pathway is mainly determined by its native topology and the iterative unfolding method can be used to reasonably describe the zinc binding process.

**Figure 6:**
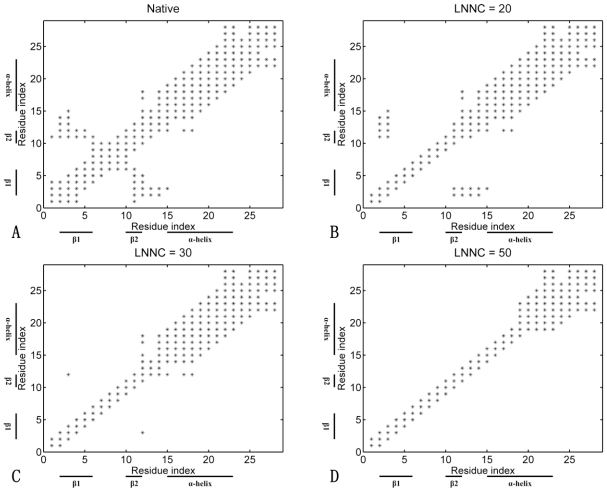
The contact maps of the native conformation (**A**), of the conformations with the LNNC to be 20 (**B**), 30 (**C**) and 50 (**D**) for FSD-1, respectively. Each native contact is marked by the symbol * in the maps.

The contact maps of the conformation in different snapshots during unfolding for FSD-1 are shown in Figure 6. This figure presents the contact maps of the native structure (Figure 6A), of the conformations with the LNNC to be 20 (Figure 6B), 30 (Figure 6C), 50 (Figure 6D) for FSD-1, respectively. The unfolding process of FSD-1 can be obtained from the change of the contact map, which is quite different from that of Sp1f2. As shown in Figure 6B, the unfolding is initiated by the partial disruption of the native contact between β-hairpin. After the loss of β-hairpin, the native contact of α-helix begins to break, which is accompanied with the disappearance of the hydrophobic core (see Figure 6 (C, D)). The previous MD simulations [23] found that in the folding process of FSD-1, the α-helix is constructed before the formation of β-hairpin. Our unfolding results also confirm this folding pathway and indicate that the α-helix is more stable compared with the β-hairpin. The previous studies [23] also found that the middle helical turn of the α-helix (R19, D20, F21 and I22) was more stable than the first helical turn (E15, K16, E17 and L18) at high temperatures. Interestingly, as shown in Figure 6 (C, D), the first helical turn is unfolding before that of the middle helical turn.

**Figure 7:**
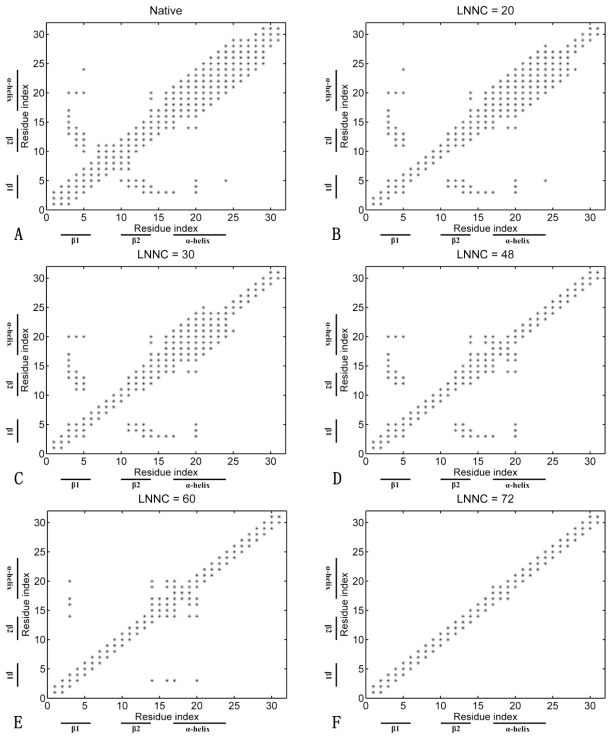
The contact maps of the native conformation (**A**), of the conformations with the LNNC to be 20 (**B**), 30 (**C**), 48 (**D**), 60 (**E**) and 72 (**F**) for Sp1f2 without zinc binding, respectively. Each native contact is marked by the symbol * in the maps.

In addition, the contact maps of unfolding snapshots for Sp1f2 without zinc binding are shown in Figure 7. Although this process is not really existing, the simulation can also help us to understand some crucial factors for the folding. As shown in Figure 7, the unfolding pathway of apo-peptide is different with those of the above two proteins. Without zinc binding, the structure of the β-hairpin is unstable. Meanwhile, the long C-terminal loop also affects the stability of α-helix, so the β-hairpin and α-helix are both unfolding in the early stage of the simulation process. As shown in Figure 7 (B–E), the native contacts of the β-hairpin disappeared faster than that with zinc binding. In contrast, the α-helix unfolded slower than that with zinc binding (see Figure 7 (B, C)). These results show that Zn(II) stabilizes the whole structure of Sp1f2. Without zinc binding, both β-hairpin and α-helix are unstable, so it is impossible for Sp1f2 to complete the correct folding.

### 3.5. Cross-Correlation Analysis during Protein Unfolding

Similar to the analysis of Su *et al*. [40,54], we explore the change of correlation between the fluctuations of residues during the unfolding process of the two proteins. The cross-correlations between the fluctuations of residues are calculated with Equation 6. The cross-correlation value ranges from −1 to 1. The positive values represent that the motions of residues are in the same direction, and the negative values represent that they move in the opposite direction. The higher the absolute cross-correlation value is, the more the two residues are correlated (or anti-correlated). On the other hand, the cross-correlation value *C* *_ij_* = 0 means that the motions of residues are completely not correlated.

**Figure 8:**
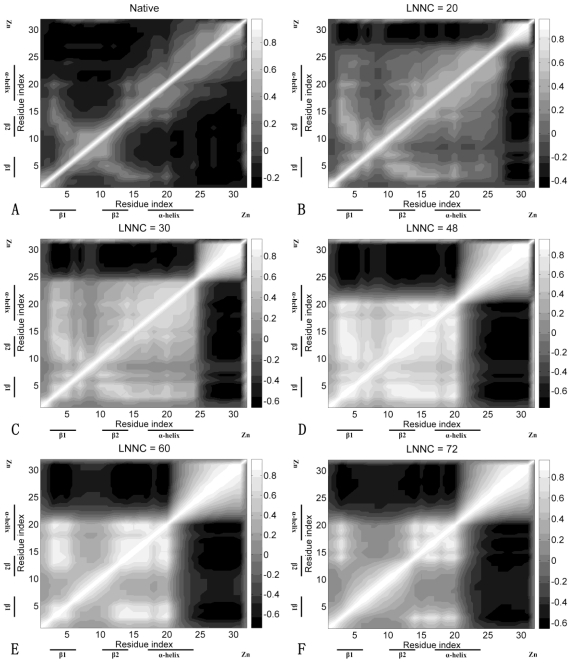
The cross-correlation maps calculated using all modes for native conformation (**A**) and several conformations with LNNC to be 20 (**B**), 30 (**C**), 48 (**D**), 60 (**E**) and 72 (**F**) during the unfolding process of Sp1f2. As shown in the bar on the right, the dark regions in the figure indicate negative correlation and the light regions present positive correlation. Both the x and y axes of the maps are residue indices.

Figure 8 presents the cross-correlation maps of Sp1f2. As shown in Figure 8A, along the diagonal of the map, there are some light blocks with positive correlations, which correspond to the secondary structures of α-helix and β-sheets. The figure also shows negative correlations between the β-hairpin and C-terminal loop. Meanwhile, the β-hairpin has higher positive correlations with Zn(II) than those of α-helix. As shown in Figure 8B, with connection of Zn(II), the β-hairpin and α-helix become a whole part and have positive correlations between them. Then, as shown in Figure 8 (C, D), with influence of C-terminal loop, the α-helix is partially unfolding and the structure of the protein seems to be divided into two parts. The β-hairpin and α-helix cooperate together and they have negative correlations with C-terminal loop. At the end of unfolding (see Figure 8 (E, F)), the β-hairpin is unfolding and only partial α-helix is kept.

**Figure 9:**
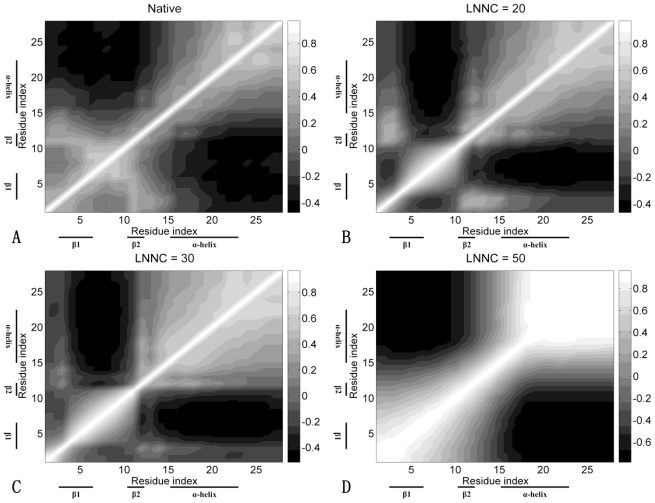
The cross-correlation maps for native conformation (**A**) and several conformations with LNNC to be 20 (**B**), 30 (**C**) and 50 (**D**) during the unfolding process of FSD-1. As shown in the bar on the right, the dark regions in the figure indicate negative correlation and the light regions present positive correlation. Both the x and y axes of the maps are residue indices.

The cross-correlation maps during the unfolding process of FSD-1 are shown in Figure 9. It is easy to identify the secondary structures and native contacts with positive correlations from the correlation map of the native structure shown in Figure 9A. As shown in Figure 9 (B, C), when the native contacts in the β-hairpin are lost, the positive correlations between the β-hairpin are reduced. Finally, the structure of the protein seems to be divided into two parts fluctuating in opposite directions (see Figure 9D). From Figure 9, we can see that in the unfolding process of FSD-1, the negative correlations between the β-hairpin and α-helix are always maintained. These phenomena show that the β-hairpin and α-helix have weak cooperativity, which is different from that of Sp1f2. These results are also consistent with previous MD simulations [22,23].

**Figure 10:**
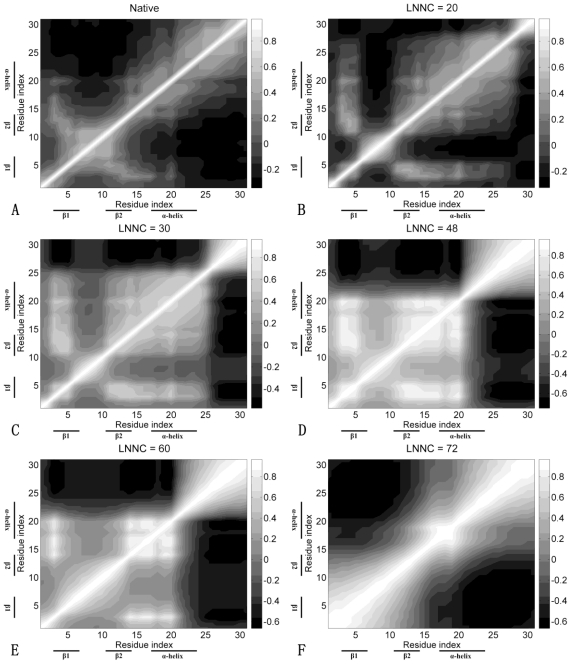
The cross-correlation maps for native conformation (**A**) and several conformations with LNNC to be 20 (**B**), 30 (**C**), 48 (**D**), 60 (**E**) and 72 (**F**) during the unfolding process of Sp1f2 without zinc binding. As shown in the bar on the right, the dark regions in the figure indicate negative correlation and the light regions present positive correlation. Both the x and y axes of the maps are residue indices.

Similar to Figure 7, the cross-correlation maps of Sp1f2 without zinc binding are also presented in Figure 10. Without zinc binding, the correlation between β-hairpin and α-helix is reduced. As shown in Figure 10 (B, C), the cross-correlation maps of apo-peptide are similar to those of FSD-1. However, because of the large fluctuation of C-terminal loop, the structure of the protein is also divided into two parts (see Figure 10 (D, E)). Then, the following cross-correlation maps are similar to those of Sp1f2 with zinc binding. The absence of zinc ion and the long C-terminal loop put the dual influences on the structure, so the cross-correlation maps of Sp1f2 without zinc binding shows the combined behaviors between those of FSD-1 and Sp1f2 with zinc binding.

### 3.6. The ANM Analysis of Sp1f2 and FSD-1

**Figure 11:**
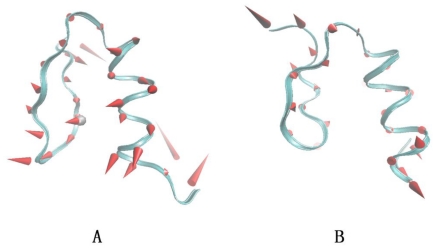
The first slowest motive mode sketch maps of the Sp1f2 (**A**) and FSD-1 (**B**). The first slow motive modes are shown with the cone model. The length of cone is correlative with the motive magnitude and the motive direction is depicted with the orientation of cone.

GNM can only provide the magnitude of displacement of atoms from their equilibrium positions for large-scale motions. To ascertain the direction of motion, ANM is applied to the structures. For the Sp1f2 structure, as seen in Figure 11A, the first slowest mode of β-hairpin and α-helix corresponds to the swing motion. However, the C-terminal loop shows an opposite direction with the other parts of the structure. The motive magnitude of the C-terminal loop is much larger than those of the other parts. It also confirms that the C-terminal loop has large fluctuation, which will affect the stability of the α-helix. As seen from Figure 11B, the first slowest motion of FSD-1 is obviously smaller than that of Sp1f2. The most flexible regions for FSD-1 are the N-terminal loop. The fluctuation of N-terminal loop will affect the stability of the β-hairpin. Compared with Sp1f2, it may explain why the α-helix is more stable compared with the β-hairpin in the FSD-1.

### 3.7. The Analysis of Folding Pathways

From the above simulations, it is found that the influence of Zn(II) on the folding of zinc-fingers lies in two points. First, Zn(II) preferentially binds to the two conserved cysteine residues and helps the formation of the β-hairpin in the early stage of folding. Second, Zn(II) makes the connection between the β-hairpin and α-helix, which increases the cooperativity of the two parts and stabilizes the whole structure of Sp1f2. On the other hand, the C-terminal loop has a negative effect for the stability of zinc-finger. In the Sp1f2, the long C-terminal loop is near to the α-helix and has large fluctuation, which results in an unfavorable effect on the formation of the α-helix. Both the Zn(II) and C-terminal loop predominate the folding pathway of the zinc-finger motif. In the folding of Sp1f2, Zn(II) accelerates the formation of the β-hairpin. Meanwhile, Zn(II) connects the β-hairpin and α-helix, and it helps the α-helix to resist the negative effect of the long C-terminal loop. Therefore, as shown in Figure 5, the folding of Sp1f2 initiates with a hydrophobic collapse. Then, with connection of Zn(II), the β-hairpin is fully formed and the α-helix is partially folded. Finally, the folding finishes with full formation of the α-helix and C-terminal loop. However, in the folding of FSD-1, the short C-terminal loop favors the stabilization of α-helix. Meanwhile, without Zn(II), the formation of β-hairpin is decelerated. Then, the folding of FSD-1 initiates with the formation of the α-helix, and it finishes with the formation of the β-hairpin (see Figure 6).

## 4. Conclusions

In this work, simple coarse-grained methods are proposed to investigate the mechanism of the zinc-finger motif. By using the Gaussian network model and anisotropy elastic network model, we analyze the binding order of Zn(II) with the conserved ligand-binding residues and the unfolding pathway of Sp1f2 and FSD-1, which are consistent with related experimental and MD simulation data. From the analysis, it is found that Zn(II) has two positive influences on the folding of zinc-fingers. On the contrary, the C-terminal loop has a negative effect on the stability of zinc-fingers. Both Zn(II) and the C-terminal loop affect the folding pathway of the different proteins. These folding and stability factors of the zinc-finger revealed in this work can provide insight into the folding mechanisms of the zinc-finger motif and help in the design of zinc-fingers. Meanwhile, these simple coarse-grained methods may be helpful for understanding the mechanisms of metal-coupled and chaperonin-assisted protein folding.
